# The role of aerosols and greenhouse gases in Sahel drought and recovery

**DOI:** 10.1007/s10584-018-2341-9

**Published:** 2018-12-07

**Authors:** Alessandra Giannini, Alexey Kaplan

**Affiliations:** 1 International Research Institute for Climate and Society, The Earth Institute at Columbia University, 61 Route 9W, Palisades, NY 10964 USA; 2 Lamont-Doherty Earth Observatory, Columbia University, 61 Route 9W, Palisades, NY 10964 USA

## Abstract

We exploit the multi-model ensemble produced by phase 5 of the Coupled Model Intercomparison Project (CMIP5) to synthesize current understanding of external forcing of Sahel rainfall change, past and future, through the lens of oceanic influence. The CMIP5 multi-model mean simulates the twentieth century evolution of Sahel rainfall, including the mid-century decline toward the driest years in the early 1980s and the partial recovery since. We exploit a physical argument linking anthropogenic emissions to the change in the temperature of the sub-tropical North Atlantic Ocean relative to the global tropical oceans to demonstrate indirect attribution of late twentieth century Sahel drought to the unique combination of aerosols and greenhouse gases that characterized the post-World War II period. The subsequent reduction in aerosol emissions around the North Atlantic that resulted from environmental legislation to curb acid rain, occurring as global tropical warming continued unabated, is consistent with the current partial recovery and with projections of future wetting. Singular Value Decomposition (SVD) applied to the above-mentioned sea surface temperature (SST) indices provides a succinct description of oceanic influence on Sahel rainfall and reveals the near-orthogonality in the influence of emissions between twentieth and twenty-first centuries: the independent effects of aerosols and greenhouse gases project on the difference of SST indices and explain past variation, while the dominance of greenhouse gases projects on their sum and explains future projection. This result challenges the assumption that because anthropogenic warming had a hand in past Sahel drought, continued warming will result in further drying. In fact, the twenty-first century dominance of greenhouse gases, unchallenged by aerosols, results in projections consistent with warming-induced strengthening of the monsoon, a response that has gained in coherence in CMIP5 compared to prior multi-model exercises.

**Electronic supplementary material:**

The online version of this article (10.1007/s10584-018-2341-9) contains supplementary material, which is available to authorized users.

## Introduction

The Sahel witnessed an outstanding climatic shift with the abrupt onset of drought in the late 1960s. The magnitude and spatial extent of the downward trend in precipitation over the twentieth century (Greene et al. 2009) and the persistence of years of deficient rainfall through the 1970s and 1980s (Lamb 1982; Nicholson 1983; Dai et al. 2004; Ali and Lebel 2009) led scientists to hypothesize that Sahelian drought may be a sign of anthropogenic change. Some pointed to the local pressure of rapid population growth on vegetation cover and the consequent impact of an increase in albedo on the atmospheric energy budget (Charney 1975). Others linked drought to the nascent preoccupation with the impact of global anthropogenic emissions, of greenhouse gases (GHGs), and other pollutants, on the general circulation of the atmosphere (Bryson 1973).

We reconcile the just-mentioned arguments about the role of anthropogenic emissions in Sahel drought, and more recent arguments attributing global precipitation changes to aerosols, natural and anthropogenic (Robock and Liu 1994; Gillett et al. 2004; Liepert et al. 2004; Lambert et al. 2004; Zhang et al. 2007; Haywood et al. 2013) with demonstration that the dominant cause of Sahel drought lies in changes in the surface temperatures of the global oceans (Folland et al. 1986; Giannini et al. 2003). We do so by linking the unique post-World War II combination of aerosol and greenhouse gas emissions to the patterns of sea surface temperature (SST) known to affect Sahel rainfall.

On interannual to millennial time scales, Sahel rainfall variations have been ascribed to interhemispheric differences in SST, whether global, restricted to the Atlantic, or to the tropical Atlantic Ocean (Lough 1986; Shanahan et al. 2009; Park et al. 2015; Lindzen and Nigam 1987; Kang et al. 2008; Schneider et al. 2014). Recent work attempting to explain the disagreement in the sign of projected twenty-first century rainfall change (Biasutti and Giannini 2006) with differences in projected SST change added the explicit consideration of oceanic warming, resulting in a linear regression model that describes Sahel rainfall as a function of two regional SST indices: the difference of tropical North and tropical South Atlantic SST averages and the average of tropical Indo-Pacific sector SSTs (Biasutti et al. 2008)*.* This regression model was further distilled into a single predictor, the North Atlantic Relative Index (NARI; Giannini et al. 2013), computed as the difference between SST averages over the sub-tropical North Atlantic (10°N–40°N, 75°W–15°W) and the global tropical oceans (20°S–20°N). Here, we denote these as NA and GT, respectively, so that NARI = NA − GT. In this difference, the tropical SST average (GT) captures global vertical stability (Chou and Neelin 2004): the stabilization effect of warming SSTs is communicated vertically through deep convection and is spread laterally by upper-atmosphere wave dynamics, given the weak temperature gradient constraint (Chiang and Sobel 2002). The sub-tropical North Atlantic SST average (NA) counters stabilization through moisture supply (Giannini et al. 2008; Seth et al. 2013). To the extent that Sahel rainfall variations on different time scales are the result of the underlying oceanic forcing’s preferred time scales of variation, our physical interpretation of NARI follows the paradigm that convection is in quasi-equilibrium with its environment (Emanuel et al. 1994). This assumption eliminates the need to consider time scales of variation separately, as long as these are longer than the time scales of convective adjustment (including the adjustment in the coupled ocean-atmosphere system described in Chiang and Sobel (2002)). In addition, it should be noted that a “relative index” effectively detrends variations (Vecchi et al. 2008), in the sense that insofar as both SST time series express GHG-induced warming, taking their difference largely removes it. In observations, NARI explains ~ 50% of the variance in twentieth century Sahel rainfall variability, all time scales included (Giannini et al. 2013).

In the “CMIP5 simulation of Sahel rainfall” section, we show that the CMIP5 multi-model mean reproduces the twentieth century evolution of Sahel rainfall, rendering discussion of its attribution to external forcing possible. In the “Climate change in the Sahel through the lens of oceanic influence” section, we propose an explanation that reconciles studies of variability, which typically seek to relate oceanic forcing and regional rainfall response, with studies of change, which typically seek to attribute regional response to external forcing, natural or anthropogenic, by developing an argument for indirect attribution of Sahel rainfall, through the influence of anthropogenic emissions on sea surface temperatures. In the “Transformation of predictors” sub-section, we exploit Singular Value Decomposition (SVD) to describe the linearly independent linear combinations of the two SST predictors defined above, i.e., the averages of sub-tropical North Atlantic and global tropical SSTs. In the “Linear regressions of Sahel rainfall” sub-section, we use the resulting singular vectors in bivariate linear regressions that describe their influence on Sahel rainfall. We present explicit formulas for these SVD-based predictors in the bivariate case; although, as noted by Preisendorfer (1988), such formulas had been available since a publication by Pearson (1901), their presentation in terms of non-dimensional parameters, introduced here, helps to visualize the underlying relationships and facilitates the interpretation of results. In particular, we show that when the ratio of standard deviations of the original predictors is close to one, the bivariate SVD rotation results in the sum and difference of these original predictor time series. In our case, this means that NARI, the difference of NA and GT, which we defined above based on a physical argument, is nearly proportional to one of the SVD predictors and is exhaustively complemented by the other, which is nearly proportional to the sum of the same SST indices, and represents global warming. The derivation of all necessary formulas is relegated to the Appendix. In the “Conclusions: Past is not prologue” section, we conclude with a synthesis that finds coherence in attributing past Sahel drought to anthropogenic emissions, while lending credence to projections of a wetter future.

## CMIP5 simulation of Sahel rainfall

The multi-model ensemble produced by the Coupled Model Intercomparison Project in support of the Fifth Assessment Report of the Intergovernmental Panel on Climate Change (IPCC), referred to as CMIP5 (Taylor et al. 2012), reproduces the observed twentieth century evolution of Sahel rainfall and projects wetter end of twenty-first century conditions more coherently than its CMIP3 predecessor (e.g., compare Biasutti and Giannini (2006) with Biasutti (2013)).

We restrict our analysis to 29 models participating in CMIP5, because only these have all the thermodynamic and dynamical variables necessary to evaluate their moisture budget (the subject of a parallel study). The models used are named in the tables in Supplementary Material. We focus on ensemble means, because we are interested in attribution of observed twentieth century Sahel rainfall variability, i.e., in describing its externally forced component, whether the external forcing is natural (i.e., due to variations in incoming top of the atmosphere insolation and spikes in aerosol concentration from volcanic eruptions) or anthropogenic (most notably, emissions of aerosols and GHGs from fossil fuel burning). Single-model ensemble sizes range between 1 and 10 in the historical simulations of the twentieth century and in the RCP8.5 scenario simulations of the twenty-first century. (In the case of the pre-Industrial control, typically, one simulation is run per model, albeit of varying length.) For each model and type of simulation, when more than one is available, we average realizations in the model’s ensemble mean. We then compute the multi-model mean as the average of the single models’ ensemble mean, a procedure which further suppresses the manifestation of internal variability.

**Fig. 1:**
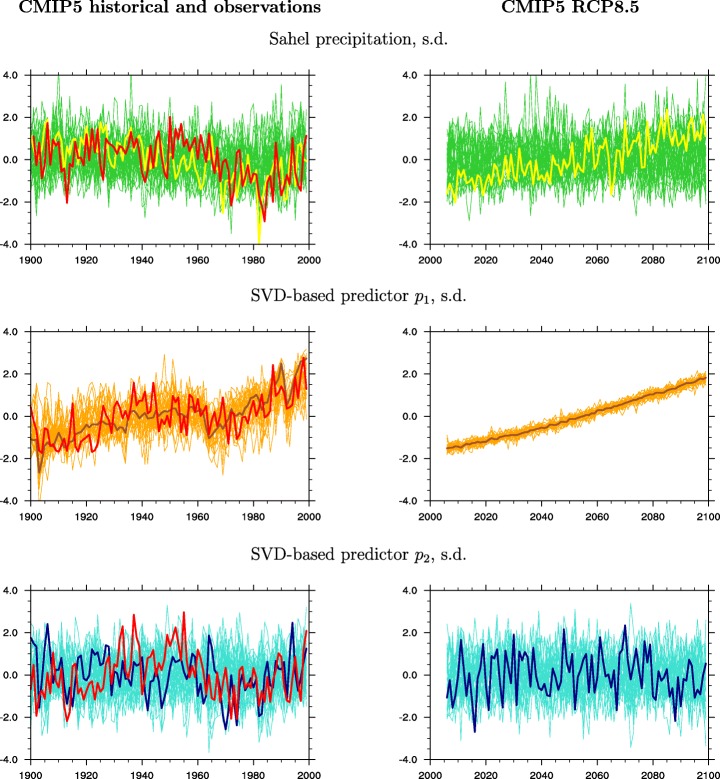
Time series of standardized Sahel rainfall (*top*) and time series of the dominant and trailing SVD modes of the predictors’ space *p*_1_ (*middle*) and *p*_2_ (*bottom*) for the single-model ensemble means of 29 CMIP5 models (*thin green*, *turquoise*, and *orange lines*) and for the multi-model ensemble mean (*thick yellow*, *brown*, and *blue lines*), from the historical simulations (1900–1999) in the *left panels*, and from the RCP8.5 simulations (2006–2099) in the *right panels*. The *red lines* represent observations

The top panels in Fig. 1 display standardized Sahel rainfall (the precipitation average for land points in 10°N−20°N, 20°W−40°E, in each model’s original resolution, over July–September (Nicholson 1983; Giannini et al. 2003)): twentieth century (1900–1999) time series are depicted on the left-hand side and twenty-first century (2006–2099) time series on the right-hand side. To facilitate comparison, we standardize all the time series: each model’s ensemble mean, in the thin green lines, is standardized by its own mean and standard deviation. The multi-model mean, in the thick yellow line, is first computed as the average of the original (non-standardized) single-model ensemble mean precipitation time series and then standardized. The reason for proceeding in this order is that we wish to characterize the externally forced component of Sahel rainfall. Since the models’ ability to capture the relevant physical processes varies, if we computed the multi-model mean on the standardized single-model ensemble means, we would give all models, “good” and “bad,” equal weight. Conversely, if the multi-model mean is taken on rainfall anomalies, the internal variability that dominates models that do not respond to external forcing is weighted out, because by definition it does not have a preferred temporal sequencing. This allows the temporally coherent response to external forcing to emerge. For a comparison to non-standardized time series, i.e., Sahel rainfall anomalies in units of mm/day, the reader is referred to Fig. 2 in Biasutti (2013): there it can be seen that (i) the amplitude of variations in the multi-model mean is smaller than that in observations, as expected, given that the single real-world realization expresses the combination of internal variability and externally forced change. However, (ii) the observed evolution falls within the envelope defined by individual ensemble member simulations. The thick red line in the left panel of our Fig. 1 represents observed Sahel precipitation (Becker et al. 2013). Its correlation with the multi-model mean is 0.37 and is corroborated by a visible qualitative match: the first half of the century is characterized by variations around the mean, and is followed by a precipitous decline that lasts until the mid-1980s, and a partial recovery since. Standardization, because it normalizes the amplitude of variation, makes it possible to clearly discern the minimum in the multi-model mean, which occurs in 1982 and coincides with the first of three driest consecutive years in observations, 1982–1984, and with the eruption of El Chichón (Robock and Liu 1994). In contrast, the twenty-first century multi-model mean (top, right panel of Fig. 1) shows an upward trend, albeit with periods characterized by interannual-to-decadal variations that mask the trend, most notably at the end of the century.

**Fig. 2:**
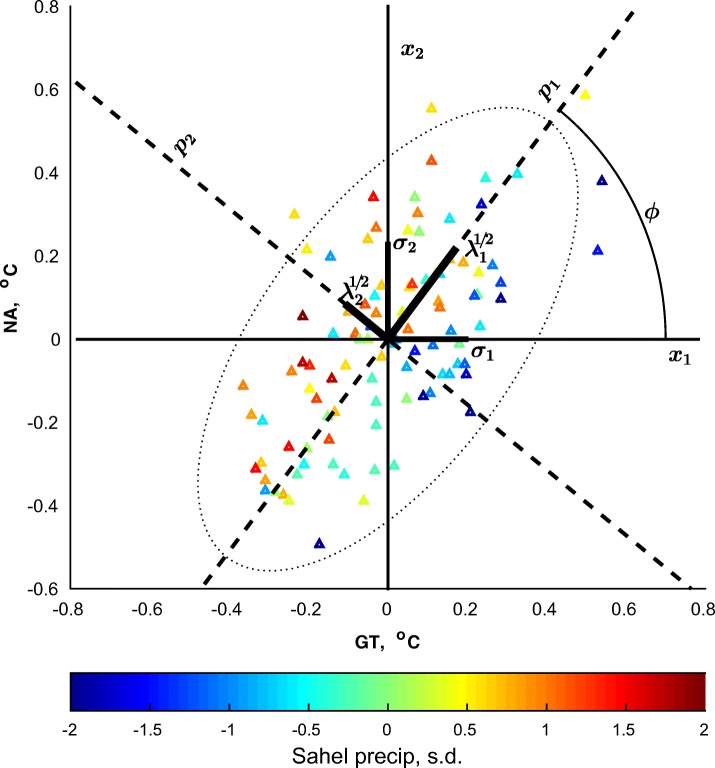
Scatterplots of sub-tropical North Atlantic and global tropical ocean temperatures in observations (1901–1999) colored by standardized values of Sahel rainfall, with depictions of the angle of rotation *ϕ*, standard deviations of the original (σ_1_, σ_2_) and SVD-based predictors (λ_1_^1/2^, λ_2_^1/2^) and the concentration ellipse (*dotted*): see text for details

## Climate change in the Sahel through the lens of oceanic influence

We build an argument for indirect attribution based on the influence of external forcing—external to the coupled ocean-atmosphere system—on the SST patterns that affect Sahel rainfall. In the “Transformation of predictors” sub-section, we concisely describe oceanic influence using SVD applied to our set of two original predictors for Sahel rainfall, sub-tropical North Atlantic and global tropical SSTs. In the “Linear regressions of Sahel rainfall” sub-section, we use the resulting singular vectors as predictors in bivariate regression. Results of the analyses of the multi-model means and observations are reported in Tables 1 and 2. Results of the analyses of the single-model ensemble means are collected in tables in Supplementary Material. The Appendices provide a brief review of the nomenclature and definitions for SVD in the general *m*-dimensional case (Singular Value Decomposition of an m-dimensional predictors’ space) and for linear regression (Use of time-dimensional singular vectors as predictors), as well as the derivation of the explicit form for the parameters of SVD in the bivariate case (Singular Value Decomposition of a two-dimensional predictors’ space; derivation of Eqs. (1)–(5)).

**Table 1:** Predictors’ space characteristics for the multi-model means from CMIP5 simulations and for twentieth century observations

Century	CMIP5 simulation	σ_1_, °C GT	σ_1_, °C NA	*ϱ*	*k*	*ϕ*, arc°	*λ*_1_ °C^2^	*λ*_2_ °C^2^	*λ*_1_/(*λ*_1_ + *λ*_2_) %	*λ*_2_/(*λ*_1_ + *λ*_2_) %
19th	PI control	0.03234	0.04195	0.369	0.771	62.75	0.0020	0.0008	71.90	28.10
20th	Historical	0.13150	0.12475	0.955	1.054	43.42	0.0321	0.0007	97.74	2.26
21st	RCP8.5	0.86711	0.83118	0.999	1.043	43.79	1.4422	0.0005	99.96	0.04
1901–1999	Observations	0.19565	0.22748	0.619	0.860	51.86	0.0734	0.0166	81.52	18.48

Columns from the left to right show the simulated century and the type of CMIP simulation (or observational reference period), the standard deviations of the original predictors, *σ*_1_ and *σ*_2_, in °C (for GT and NA, respectively), their correlation coefficient, *ρ*, the ratio of their standard deviations, *k* = *σ*_1_/*σ*_2_, angle *ϕ* from Eq. (3), in arc degree, variances *λ*_1_ and *λ*_2_, in (°C)^2^, respectively, explained by the leading and trailing modes, corresponding to time series *p*_1_ and *p*_2_, and the same in percent of the total variance *λ*_1_ + *λ*_2_

**Table 2:** Regression coefficients and skill for Sahel rainfall in CMIP5 multi-model means and in twentieth century observations

Century	CMIP5 simulation	*a*	*b*	Correlation coefficient of *y*, *ŷ*
19th	PI-control	0.054	0.312	0.317
20th	Historical	−0.320	0.477	0.574
21st	RCP8.5	0.783	0.171	0.801
1901–1999	Observations	−0.182	0.607	0.633

Columns from the left to right show the simulated century and the type of CMIP simulation (or observational reference period), regression coefficients *a* and *b* of Sahel rainfall on the time series *p*_1_ and *p*_2_, respectively, and the correlation coefficient between *y*, Sahel rainfall simulated in CMIP5, or observed, and its predicted values from regression model (6): *ŷ* = *ap*_1_ + *bp*_2_

### Transformation of predictors

We relate differences between our predictors’ covariance structures to differences in the external forcing applied to the pre-Industrial control and twentieth and twenty-first century simulations. The latter two types of simulations are run with time-varying external forcing, natural and anthropogenic: variations in each realization of a single model’s ensemble are a combination of internal variability and externally forced change, and their ensemble mean is taken to filter out internal variability. The former are run with constant external forcing, including CO_2_ concentrations held fixed at pre-Industrial levels (280 ppm), the intent being to provide a truthful estimation of each model’s internal variability. We repeat analyses on each model’s first 100 years of the pre-Industrial control simulation (typically, there is only one such simulation per model, of variable length) and on each model’s ensemble mean (with ensemble sizes varying between one and ten) for the twentieth and twenty-first century simulations.

When there are only two predictors—in our case, *x*_1_ = GT and *x*_2_ = NA—standardized and mutually uncorrelated SVD-based predictors *p*_1_, *p*_2_ are obtained from the original predictors *x*_1_, *x*_2_ by orthogonal rotation in the *x*_1_*x*_2_ plane followed by rescaling:

1 $$ {p}_1=\left({x}_1\cos \phi +{x}_2\sin \phi \right)/\sqrt{\lambda_1} $$

and

2 $$ {p}_2=\left({x}_2\cos \phi -{x}_1\sin \phi \right)/\sqrt{\lambda_2} $$

where the rotation angle *ϕ* and squared rescaling coefficient *λ*_1_, *λ*_2_ are given by:

3 $$ \phi =\Big\{{\displaystyle \begin{array}{cc}\frac{1}{2}\operatorname{arccot}\left[\left(k-{k}^{-1}\right)/2\rho \right]& :\rho >0\\ {}\pi /2& :\rho =0,0<k<1\\ {}0& :\rho =0,k\ge 1\\ {}\frac{1}{2}\operatorname{arccot}\left[\left(k-{k}^{-1}\right)/2\rho \right]+\pi /2& :\rho <0\end{array}} $$

and

4 $$ {\lambda}_1=\frac{1}{2}\left({\sigma}_1^2+{\sigma}_2^2+\mathcal{D}\right),{\lambda}_2=\frac{1}{2}\left({\sigma}_1^2+{\sigma}_2^2-\mathcal{D}\right),\mathcal{D}={\left[{\left({\sigma}_1^2-{\sigma}_2^2\right)}^2+4{\rho}^2{\sigma}_1^2{\sigma}_2^2\right]}^{1/2}. $$

In Eqs. (3)–(4), *k* = *σ*_1_/*σ*_2_ is the ratio of standard deviations σ_1_ and σ_2_ of *x*_1_ and *x*_2_, respectively, and *ρ* is their correlation coefficient. Incidentally, *k* and *ρ* are the two non-dimensional parameters, characterizing the variance-covariance matrix of *x*_1_ and *x*_2_ up to a constant factor; to represent the same in terms of *p*_1_ and *p*_2_, one such parameter is *ϕ*, while the relative value of either of *λ*_1_ or *λ*_2_ where *λ*_1_ > *λ*_2_, can be another:

5 $$ \frac{\lambda_2}{\lambda_1+{\lambda}_2}=\frac{1}{2}-\frac{1}{2}{\left[{\left(\frac{k-{k}^{-1}}{k+{k}^{-1}}\right)}^2+{\left(\frac{2\rho }{k+{k}^{-1}}\right)}^2\right]}^{1/2}. $$

Derivation of Eqs. (1)–(5) is given in “Singular Value Decomposition of a two-dimensional predictors’ space; derivation of Eqs. (1)–(5)”.

Figure 2 illustrates these relationships in twentieth century observations (see the last row of Table 1 for the values of relevant parameters). The observed values of the original predictors are the coordinates of the color triangles on the (*x*_1_, *x*_2_) plane, with the corresponding standardized values of Sahel rainfall shown by color. The thick segments shown on the *x*_1_, *x*_2_ axes indicate sample standard deviations, *σ*_1_ and *σ*_2_, of these original predictors. Their values are approximately 0.20 and 0.23 °C, respectively, and their ratio is *k* = *σ*_1_/*σ*_2_ = 0.86. The correlation coefficient of the original predictors is *ρ* = 0.62. SVD-based predictors *p*_1_, *p*_2_, whose corresponding axes are shown by dash lines, are obtained by orthogonal rotation of the original predictors *x*_1_, *x*_2_ with rotation angle about ϕ = 52°, indicated by the arc connecting the *x*_1_ and *p*_1_ axes. The thick segments on the *p*_1_ and *p*_2_ axes show standard deviations (in our notation, *λ*_1_^1/2^ = 0.27 °C and *λ*_2_^1/2^ = 0.13 °C) of the data projections on these axes. The variance of projections is maximized for *p*_1_, among all possible directions on the *x*_1_, *x*_2_ plane, therefore *λ*_1_ > *λ*_2_, necessarily. Also by construction, the in-sample correlation coefficient between *p*_1_ and *p*_2_ is zero; since there are only two predictors, *p*_2_, being the “last” of them, minimizes the variance among all directions on the *x*_1_, *x*_2_ plane. Therefore, *p*_2_ only explains *λ*_2_/(*λ*_1_ + *λ*_2_) = 18.5% of variance in the sample (while *p*_1_ explains the other 81.5%). These features of the data and predictors’ pairs are illustrated by the shape of “concentration ellipses” (von Storch and Zwiers 1999; see their example 2.8.12, p. 43). The 95% concentration ellipse is shown by the dotted line in Fig. 2; its major semiaxes are 2.45*λ*_1_^1/2^ and 2.45*λ*_2_^1/2^. It should be noted that *λ*_1_ and *λ*_2_ do not necessarily determine the relative importance of the predictors *p*_1_ and *p*_2_ in modeling other variables: as color changes among triangles in Fig. 2 demonstrate, *p*_2_ is much more important than *p*_1_ in describing Sahel rainfall changes over 1901–1999. This is confirmed by the calculation of the regression coefficients in the last row of Table 2.

**Fig. 3:**
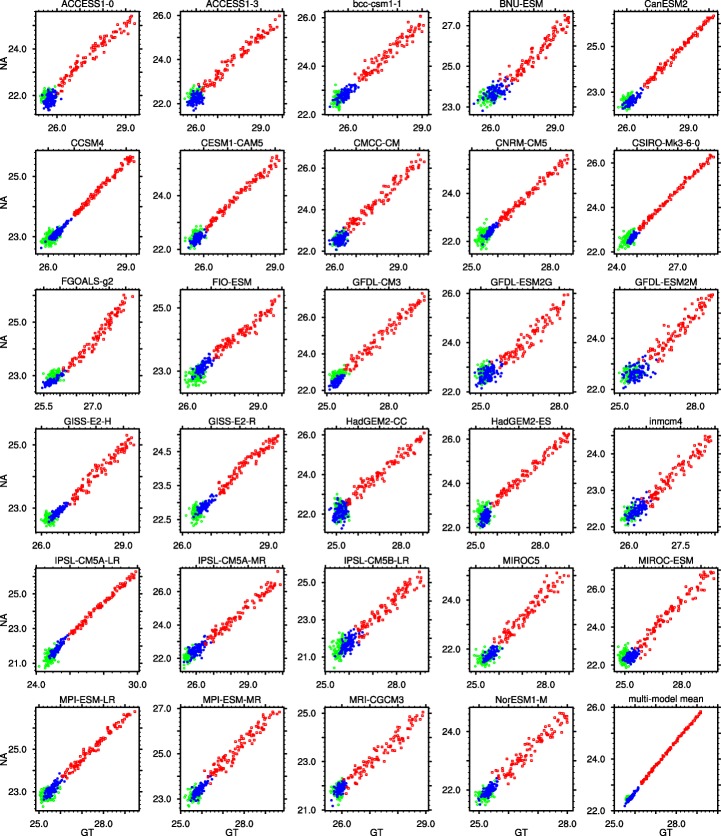
Scatterplots of sub-tropical North Atlantic and global tropical ocean temperatures in 100 years of pre-Industrial control (*green circles*), in the twentieth century/historical (*blue dots*), and in the twenty-first century/RCP8.5 (*red squares*) simulations. The first 29 panels correspond to single-model ensemble means, and the last panel (in the lower right corner) corresponds to the multi-model mean

In models, since the ratio, *k*, of standard deviations of global tropical and sub-tropical North Atlantic SSTs is also close to 1 in the twentieth and twenty-first centuries, and the correlation coefficient of the SST indices, *ρ*, is positive (Table 1), the angle *ϕ* in Eq. (3) is close to 45°. That the diagonal is the direction along which is expressed the greatest covariance between *x*_1_ = GT and *x*_2_ = NA can also be clearly seen in Fig. 3. The remaining variance, orthogonal to it by construction, is larger in the twentieth (blue dots in Fig. 3) than in the twenty-first century (red squares). Since sin*ϕ* = cos*ϕ* for *ϕ* = 45^o^, *p*_1_ and *p*_2_, respectively, in Eqs. (1) and (2), become nearly proportional to the sum and difference of the original predictors, and specifically, *p*_2_ approximates NARI. The time series of *p*_1_ are depicted in the middle panels of Fig. 1: indeed, *p*_**1**_ represents a “global warming” mode, evident in both sub-tropical North Atlantic and global tropical ocean temperatures. *p*_2_ (~NARI) explains more variance in the twentieth than in the twenty-first century. Its standardized form is depicted in the bottom panels of Fig. 1. In the middle and bottom left-hand side panels, thick red lines represent the principal components in observations (Kaplan et al. 1998).

Our original predictors, GT and NA, are highly correlated in the twentieth and twenty-first century simulations, less so in the pre-Industrial control simulations (Fig. 3 and Table 1). As a consequence, the percent of variance explained by the leading mode, *λ*_1_ in Eq. (4), increases in the simulations with time-dependent external forcing, as compared to those with constant forcing. The correlation further increases from twentieth to twenty-first century and consequently so does the relative variance of the leading mode, *λ*_1_/(*λ*_1_ + *λ*_2_) = 1 − *λ*_2_/(*λ*_1_ + *λ*_2_), see Eq. (5). This behavior represents one prominent way in which external forcing expresses itself: it forces a positive correlation between the two predictors, through their common, GHG-induced warming. In addition, the relative cooling of the North Atlantic associated with aerosols contributes to the co-variability of indices with a second, preferred direction along which variance is expressed, which by construction is orthogonal to the first. This direction expresses more variance, visible in the scatter plots in Fig. 3, in the twentieth (blue dots) than in the twenty-first century (red squares).

### Linear regressions of Sahel rainfall

Our SVD analysis of the original predictors confines any trend entirely to the leading mode time series, *p*_1_, i.e., the “sum” or “warming” mode, while the residual mode, *p*_2_, i.e., the “difference” or NARI, is completely devoid of any significant trend. We model standardized Sahel rainfall *y*, our predictand, by linear regression on *p*_1_ and *p*_2_ (see Section “Use of time-dimensional singular vectors as predictors” in the Appendix):

6 $$ \widehat{y}=a{p}_1+b{p}_2 $$

Regression coefficients *a* and *b* and the correlation coefficients between simulated and predicted Sahel rainfall for the multi-model mean and for observations are reported in Table 2. Since the SVD-based predictor time series are standardized and mutually uncorrelated, and predictand time series are standardized, the regression coefficients, *a* and *b*, are precisely the correlation coefficients of the predictand and corresponding predictors. In the twentieth century, correlation of the multi-model mean simulation of Sahel rainfall with its SST-based regression is 0.574 and correlation in observations is 0.633. Both terms in the regression contribute, but *p*_2_ contributes more (since *b > a*): late twentieth century Sahel drought is explained by the absence of North Atlantic warming, relative to warming of the global tropical oceans. The (positive) sign of *b*, the regression coefficient multiplying *p*_2_ (NARI), is consistent across centuries. However, its magnitude and explanatory power vis à vis Sahel rainfall are drastically diminished in the twenty-first century. Over the twenty-first century, the correlation between the simulated Sahel rainfall and its in-sample prediction is 0.801. The multi-model ensemble achieves this high a correlation essentially through a positive correlation between Sahel rainfall and warming, captured by *p*_1_. This happens because the coherence among models in projections of twenty-first century precipitation change has increased considerably from CMIP3 to CMIP5 (Biasutti 2013): a majority of models in CMIP5 project a future increase in Sahel rainfal to go along with warming (Schewe and Levermann 2017), in analogy to paleoclimate reconstructions of the “Green Sahara”, when summer insolation was stronger than now (de Menocal et al. 2000). Among the models with a twenty-first century correlation coefficient between *p*_1_ and Sahel rainfall larger than 0.45 (thus explaining ~ 20% of the variance with the single predictor *p*_1_), seven of nine project future wetting (Table S3 in Supplementary Materials). In sum, regression models between centuries are nearly orthogonal: *p*_2_, analogous to the difference of predictors or NARI, explains a larger fraction of twentieth century variation, while *p*_1_, analogous to the sum of predictors or warming, alone explains the externally forced twenty-first century change.

## Conclusions: Past is not prologue

Our analysis of the multi-model mean by design emphasizes the externally forced response and leads us to propose the following indirect attribution argument for Sahelian climate change, past and future. In the second half of the twentieth century, as global dimming (Stanhill and Cohen 2001; Liepert 2002) opposed global warming in the northern hemisphere, a unique combination of anthropogenic emissions contributed to the late twentieth century drying of the Sahel through their effect on sea surface temperatures: aerosols by cooling the North Atlantic and greenhouse gases by warming the tropical oceans, especially the Indian Ocean. Warming of the global tropical oceans “upped the ante” for deep convection (Chou and Neelin 2004; Held et al. 2005), while the absence of warming in the North Atlantic reduced the moisture supply to the monsoon and thus its  potential to meet the “upped ante” and to trigger vertical instability (Giannini et al. 2008). Our argument is distinct from others previously proposed, which attributed late twentieth century Sahel drought solely to aerosols, whether through cooling of the North Atlantic or of the entire northern hemisphere (Rotstayn and Lohmann 2002; Kawase et al. 2010; Ackerley et al. 2011; Booth et al. 2012; Hwang et al. 2013*;* Park et al. 2015; Wang et al. 2016), in three ways. First of all, we argue for an *indirect effect* of anthropogenic emissions, i.e., mediated by sea surface temperatures. Linking emissions to Sahel rainfall through SSTs has the added advantage that it allows the synthesis in a single physical explanation of *natural* variability and *anthropogenic* change. Secondly, our argument is not about the role of interhemispheric gradients in SST shifting the latitudinal location of the Intertropical Convergence Zone and by extension rainfall in the Sahel. Rather, it is about *quasi-equilibrium in convection* as the world warms. The observed late twentieth century drying of the Sahel was more profound—longer lasting and of greater amplitude—than, e.g., the early twentieth century drought around 1910, despite the North Atlantic being cooler during the early period. This observation justifies the search for additional factors in drought, which we identify in greenhouse gas-induced tropical warming, which in the twentieth century occurred simultaneously with North Atlantic cooling. Therefore, thirdly, we argue for the *combined drying effect* of the two contrasting anthropogenic influences in twentieth century: drought was not caused by aerosols alone or by the cooling of the North Atlantic alone. Neither could it have been caused by greenhouse gases alone, as is made evident in simulations which include only the latter forcing (Biasutti 2013; Dong and Sutton 2015; Gaetani et al. 2017; Hill et al. 2017). Rather, drought resulted from the combination of aerosols and greenhouse gases. One influence cooled and reduced the moisture supply, while the other, warming, raised the threshold for convection—a double jeopardy. In the twenty-first century, external influence is dominated by GHG-induced warming, of both the North Atlantic and global tropical oceans. NARI, their difference, tends to zero, leaving all response to external forcing to be explained by their sum.

SVD rotation of the original predictors to their weighted sum and difference and the relative sizes of their regression coefficients (Table 2) expose the near-orthogonality of the twentieth and twenty-first centuries in the response of SSTs and rainfall to external forcing. The oceans’ translation of external forcing of predominantly anthropogenic nature leads to contrasting but not inconsistent outcomes in the past and the future. Late twentieth century drying and twenty-first century projections of wetting are consistent with different balances in the influences of aerosol and greenhouse gas emissions on regional energy and moisture budgets, underlined by one coherent physical explanation rooted in the dynamics of quasi-equilibrium in convection. The fact that emissions need to be considered holistically, not additively, explains how it is possible that while anthropogenic warming had a role in past drought, continued warming in the twenty-first century gives rise to a strengthening of the monsoon. As aerosol emissions have abated, especially around the North Atlantic, it is the combination of warmings of both North Atlantic and global tropical oceans that explains the strengthening of the monsoon: the upped ante can now be met. This situation is consistent with understanding of monsoon response to warming on paleoclimate time scales: when the ocean warms enough to contribute to an “upped ante” with increased moisture supply but not enough to shift the favored locus of convection from land to ocean (Chou et al. 2001), it reinforces the monsoon, as is the case for West Africa (Braconnot et al. 2012). With the usual caveats related to uncertainty in multi-model mean projections, that further warming may result in wetting of the Sahel is a conclusion worth reinforcing, in equal parts because it has gained in coherence between CMIP3 and CMIP5 (Biasutti 2013), and because the recovery of the rains is being experienced on the ground, in a fashion that is remarkably consistent with expectation from anthropogenic warming (West et al. 2008; Lodoun et al. 2013).

### Electronic supplementary material

ESM 1 (PDF 98.1 kb)

## Figures and Tables

**Fig. 4:**
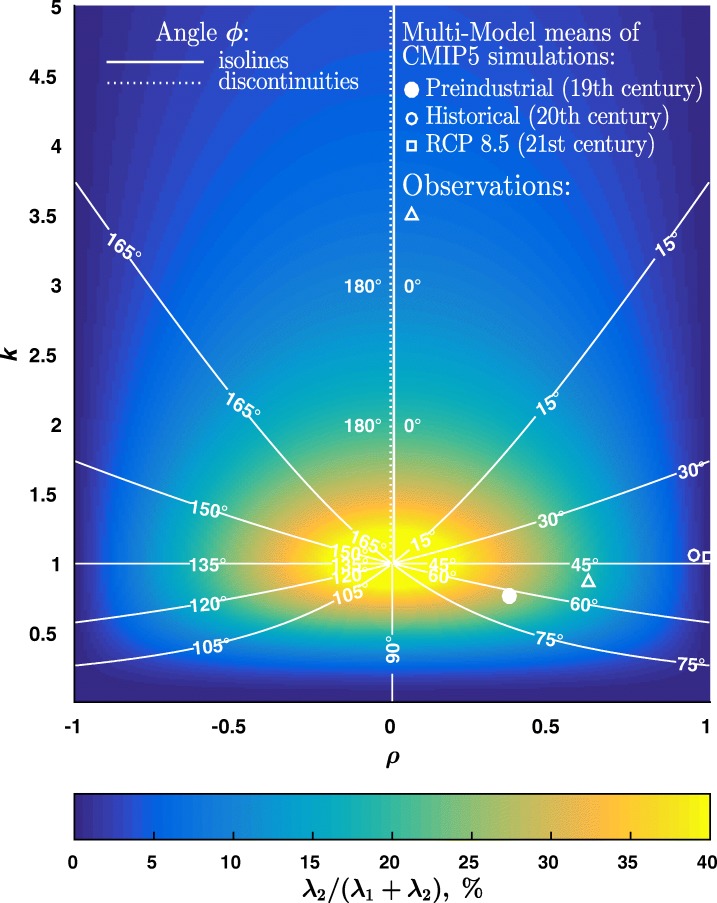
Dependence of the angle *ϕ* (*white contours*) and of ratio *λ*_2_/(*λ*_1_ + *λ*_2_), in percent (*color shading*) on the correlation coefficient *ρ* and ratio of standard deviations *k* of GT and NA. White dot, circle, square, and triangle show points on the (*ρ, k*) plane corresponding to the multi-model means of CMIP5 pre-Industrial control, historical, and RCP8.5 scenario simulations and of observations, respectively. Note that ϕ(*ρ*, *k*) is discontinuous at all points of the ray {(*ρ*, *k*) : *ρ* = 0, *k* ≥ 1}. In particular, while the definition in Eq. (3) made *ϕ* right-continuous at *ρ* = 0 for all *k* > 1, since ϕ(0, *k*) = ϕ(0+, *k*) = 0, the left limit of *ϕ* in these points is different: ϕ(0−, *k*) = *π*

## Appendix. Methods

### Singular Value Decomposition of an *m*-dimensional predictors’ space

Suppose an *n* × *m* matrix **X** contains, by columns, centralized samples of *m* variables *x*_1_, *x*_2_, ..., *x*_m_ (our *original predictors*), each observed at the same *n > m* times. The well-known *thin* Singular Value Decomposition (SVD) presents **X** as:

$$ \mathbf{X}=\mathbf{U}\boldsymbol{\Sigma } {\mathbf{E}}^{\mathbf{T}} $$

where **U** is an *n* × *m* matrix of orthonormal columns, **E** is an *m* × *m* orthogonal matrix, and **Σ** is a diagonal matrix with non-negative diagonal elements in non-ascending order, called singular values (Golub and Van Loan 1996). Superscript ^*T*^ denotes matrix transposition. The columns of **U** and **E** are called left and right singular vectors. Rescaling the left singular vectors by $\mathbf{P}=\sqrt{n-1}\mathbf{U}$ and introducing **Λ** = **Σ**^2^/(*n* − 1), we rewrite **X** as:

7 $$ \mathbf{X}=\mathbf{P}{\boldsymbol{\Lambda}}^{1/2}{\mathbf{E}}^{\mathrm{T}} $$

By construction, the columns of **P** are standardized, mutually uncorrelated time series. Each column is associated with a pattern of *loadings* (from the corresponding column of matrix **E**) on the original *m* variables and with the amount of variance (given by the corresponding diagonal element in **Λ**) that it *explains* within the total variance of the *m* original variables. The latter is easy to see from observing that the variance-covariance matrix of the original variables:

8 $$ \mathbf{C}={\mathbf{X}}^{\mathrm{T}}\mathbf{X}/\left(n-1\right)=\mathbf{E}\boldsymbol{\Lambda } {\mathbf{E}}^{\mathrm{T}} $$

has its eigenvalues and eigenvectors equal to diagonal elements of **Λ** and to columns of **E**, respectively. The columns of **E** are also known as Empirical Orthogonal Functions (EOFs), while the columns of **V** = **PΛ**^1/2^ are known as Principal Components (PC) of the data set contained in matrix **X**. Decomposition of the data set into EOFs and PCs:

$$ \mathbf{X}={\mathbf{VE}}^{\mathrm{T}} $$

is often used in climate science to represent the overall co-variability of the original variables as the superposition of linearly independent modes, with several leading modes usually explaining most of the total variance in the system (von Storch and Zwiers 1999).

### Singular Value Decomposition of a bivariate predictors’ space; derivation of Eqs. (1)–(5)

We centralize our predictors, denote them *x*_**1**_ and *x*_**2**_, respectively, arrange them column-wise in matrix **X** = [**x**_1_**x**_2_], and perform the SVD of **X** in the form of Eq. (7). The columns of matrix **P** = [**p**_1_ **p**_2_], called singular vectors, are obtained from the columns of **X** by an orthogonal rotation with 2x2 matrix **E**, followed by their normalization by the square roots of diagonal elements of 2x2 matrix **Λ**:

9 $$ \mathbf{P}=\mathbf{XE}{\boldsymbol{\Lambda}}^{-1/2} $$

By construction, **p**_1_ and **p**_2_ are standardized, mutually uncorrelated time series.

When there are only two predictors, *x*_1_ and *x*_2_, it is possible to present the elements of matrices **E** and **Λ** of the canonical decomposition of the predictors’ variance-covariance matrix **C** = **X**^T^**X**/(*n* − 1) = **EΛE**^T^ as functions of its elements.

Let standard deviations of *x*_1_ and *x*_2_ be *σ*_1_ and *σ*_2_, respectively, and their correlation coefficient be *ρ*, then their variance-covariance matrix is:

10 $$ \mathbf{C}=\left[\begin{array}{cc}{\sigma}_1^2& \rho {\sigma}_1{\sigma}_2\\ {}\rho {\sigma}_1{\sigma}_2& {\sigma}_2^2\end{array}\right]. $$

Note that:

$$ \mathbf{C}=\frac{\sigma_1^2+{\sigma}_2^2}{2}\ \mathbf{I}+{\left[{\left(\frac{\sigma_1^2+{\sigma}_2^2}{2}\right)}^2+{\rho}^2{\sigma}_1^2{\sigma}_2^2\right]}^{1/2}\left[\begin{array}{cc}\cos 2\phi & \sin 2\phi \\ {}\sin 2\phi & -\cos 2\phi \end{array}\right], $$

where **I** is a 2x2 identity matrix, and *ϕ* from the interval [0, *π*) is determined by:

11 $$ \cot \left(2\phi \right)=\left({\sigma}_1^2-{\sigma}_2^2\right)/\left(2\rho {\sigma}_1{\sigma}_2\right). $$

Introducing a non-dimensional parameter *k* = *σ*_1_/*σ*_2_, and expressing *ϕ* from Eq. (11) as a function of *k* and *ρ*, we obtain Eq. (3).

Using the identity:

$$ \left[\begin{array}{cc}\;\cos 2\phi & \sin 2\phi \\ {}\sin 2\phi & -\cos 2\phi \end{array}\right]=\left[\begin{array}{cc}\;\cos \phi & -\sin \phi \\ {}\sin \phi & \kern0.24em \cos \phi \end{array}\right]\left[\begin{array}{cc}1& 0\\ {}0& -1\end{array}\right]{\left[\begin{array}{cc}\cos \phi & -\sin \phi \\ {}\sin \phi & \cos \phi \end{array}\right]}^T $$

we rewrite:

$$ \mathbf{C}=\left[\begin{array}{cc}\cos \phi & -\sin \phi \\ {}\sin \phi & \cos \phi \end{array}\right]\left[\begin{array}{cc}{\lambda}_1& 0\\ {}0& {\lambda}_2\end{array}\right]{\left[\begin{array}{cc}\cos \phi & -\sin \phi \\ {}\sin \phi & \cos \phi \end{array}\right]}^T $$

with *λ*_1_ and *λ*_2_ defined by Eqs. (4). Therefore, the canonical decomposition of **C** is performed with eigenvector and eigenvalue matrices, **E** and **Λ**, as follows:

12 $$ \mathrm{E}=\left[\begin{array}{cc}\cos \phi & -\sin \phi \\ {}\sin \phi & \cos \phi \end{array}\right],\kern0.5em \boldsymbol{\Lambda} =\left[\begin{array}{cc}{\lambda}_1& 0\\ {}0& {\lambda}_2\end{array}\right] $$

where *ϕ* is given by Eq. (3) and *λ*_1_ and *λ*_2_ by Eq. (4).

Inserting expressions for **E** and **Λ** from Eq. (12) into Eq. (9), we write them out in the component form for an arbitrary row of matrix P to obtain Eqs. (1)–(2).

Preisendorfer (1988) noted that Eqs. (11) and (4) are known since Pearson (1901). Equation (3) derived above gives an explicit form for the angle *ϕ*, expressing it through the non-dimensional parameters *k* and *ρ* of the original predictors’ variance-covariance matrix, making it more convenient for interpretation. A traditional non-dimensional parameter of such matrix for SVD-based predictors *p*_1_, *p*_2_, the portion of variance explained by one of the predictors, follows from Eq. (4). For *p*_1_, it is expressed through *k* and *ρ* as:

13 $$ \frac{\lambda_1}{\lambda_1+{\lambda}_2}=\frac{1}{2}+\frac{1}{2}{\left[{\left(\frac{k-{k}^{-1}}{k+{k}^{-1}}\right)}^2+{\left(\frac{2\rho }{k+{k}^{-1}}\right)}^2\right]}^{1/2}. $$

For the predictor *p*_2_ with the smaller variance, *λ*_2_, since *λ*_2_/(*λ*_1_ + *λ*_2_) = 1 − *λ*_1_/(*λ*_1_ + *λ*_2_), using (13) results in Eq. (5). The general structure of the dependence of the angle *ϕ*, characterizing the rotation of the original predictors toward uncorrelated singular vectors, and of ratio *λ*_2_/(*λ*_1_ + *λ*_2_), characterizing the relative variance of the second (trailing) predictors’ mode, on non-dimensional parameters of the original predictors’ variance-covariance matrix, *ρ*, the correlation coefficient of the original predictors *x*_1_ and *x*_2_, and *k*, their ratio of standard deviations, are illustrated in Fig. 4, with points corresponding to the data samples of multi-model means from CMIP5 simulations and from observations, as listed in Table 1.

Finally, the relationships between parameters of variance-covariance matrices in the (*x*_1_, *x*_2_) and (*p*_1_, *p*_2_) predictor planes are illustrated by the shape of “concentration ellipses,” which are described by equation:

$$ {x}_1^2/{\sigma}_1^2-2\rho {x}_1{x}_2/\left({\sigma}_1{\sigma}_2\right)+{x}_2^2/{\sigma}_2^2=\left(1-{\rho}^2\right)q\left(\alpha \right) $$

in (*x*_1_, *x*_2_) coordinates and by equation:

$$ {p}_1^2/{\lambda}_1+{p}_2^2/{\lambda}_2=q\left(\alpha \right) $$

in (*p*_1_, *p*_2_) coordinates. Here, *q*(*α*) is the *α*% quantile of the *χ*^2^(2) distribution with two degrees of freedom, so that if (*x*_1_, *x*_2_) observations obey a bivariate normal distribution, *α*% of the data points is contained within this concentration ellipse (von Storch and Zwiers 1999, see their example 2.8.12, p. 43). An example of such an ellipse, expected to contain (for normally distributed data) *α* = 95% of the data, is shown in Fig. 2.

### Use of time-dimensional singular vectors as predictors

In regression studies, it often proves advantageous to use as predictors a set of standardized and mutually uncorrelated variables, like *p*_1_, *p*_2_, ..., *p*_*m*_ (their time series **p**_i_, *i* = 1, 2, ..., *m*, are given by columns of matrix **P** and represent rescaled time-dimensional (left) singular vectors of **X**), instead of the original variables that lack these properties, leading to more tractable and easier to interpret results. Indeed, consider the regression model:

14 $$ y={a}_1{p}_1+{a}_2{p}_2+\dots +{a}_m{p}_m+\varepsilon $$

where *ε* is a random error and, for simplicity, the predictand *y* is assumed standardized. The vector of unknown regression coefficients **a** = (*a*_1_, *a*_2_, …, *a*_*m*_)^*T*^ has to be found by the least-squares best fit to the system

$$ \mathbf{y}=\mathbf{Pa} $$

where **y** is an *n*-dimensional vector—a column that contains the standardized data sample for variable *y*. Dividing normal equations

$$ \left({\mathbf{P}}^{\mathrm{T}}\mathbf{P}\right)\mathbf{a}={\mathbf{P}}^{\mathrm{T}}\mathbf{y} $$

by *n*–1, we find, since **P**^T^**P**/(*n* −1) = **I** (where **I** is an *n* × *n* identity matrix),

$$ \mathbf{a}={\mathbf{P}}^{\mathrm{T}}\mathbf{y}/\left(n-1\right)={\left({\rho}_1,{\rho}_2,\dots, {\rho}_m\right)}^T, $$

where *ρ*_*i*_, for each *i* = 1, 2, ..., *m*, is the sample correlation coefficient between *p*_i_ and *y* (${\rho}_i={p}_i^Ty/\left(n-1\right)$, since **y** and columns of **P** are all standardized). Therefore, regression model Eq. (14) results in the prediction:

15 $$ \widehat{y}={\rho}_1{p}_1+{\rho}_2{p}_2+\dots +{\rho}_m{p}_m $$

for which the sample estimate $\widehat{\rho}$ of its correlation coefficient with the actual value of *y* satisfies the relationship:

$$ {\widehat{\rho}}^2={\rho}_1^2+{\rho}_2^2+\dots {\rho}_m^2, $$

making it easy to see how the inclusion of time series *p*_*i*_ of each individual mode *i* into the regression model contributes to the overall skill of prediction. Another measure of in-sample skill estimate for prediction Eq. (15), the standard deviation $\widehat{e}$, of the difference $\hat{y}-y$, can be expressed through $\widehat{\rho}$ as $\widehat{e}=\sqrt{1-{\widehat{\rho}}^2}$.
