# Supplementary material for: The role of aerosols and greenhouse gases in Sahel drought and recovery
Source: Clim Change. 2018 Dec 7;152(3):449–66. doi: 10.1007/s10584-018-2341-9 (PMC6445402; doi:10.1007/s10584-018-2341-9)
Supplement: Supplementary file 1 — (PDF 98.1 kb) [file 10584_2018_2341_MOESM1_ESM.pdf]

Climatic Change manuscript No.
(will be inserted by the editor)

The role of aerosols and greenhouse gases in Sahel drought and recovery: Supplementary material

Alessandra Giannini · Alexey Kaplan

Received: 27 May 2018 / Accepted: 12 November 2018

A. Giannini
International Research Institute for Climate and Society, Earth Institute at Columbia University, 61 Rt 9W, Palisades NY 10964, U.S.A.

A. Kaplan
Lamont-Doherty Earth Observatory, Earth Institute at Columbia University, 61 Rt 9W, Palisades NY 10964, U.S.A.

Supplementary Material

Table S1: Predictors' space characteristics and linear regression results for Sahel rainfall from each individual model's ensemble mean in CMIP5 pre-Industrial control simulations. Columns from left to right show model name, the correlation coefficient, ρ, of the original predictors (x_1 =GT, global tropical SST mean, and x_2 =NA, sub-tropical North Atlantic SST mean—see Section 1 for the exact definition of these indices), the ratio of their standard deviations, $k = \sigma_1/\sigma_2$, angle ϕ from Eq. (3), in arc degree, the relative variance of the trailing mode, $\lambda_2/(\lambda_1 + \lambda_2)$, bivariate regression coefficients *a* and *b* of Sahel rainfall *y* (see Section 2 for an exact definition) on the SVD-based predictors p_1 and p_2, obtained from the original predictors x_1 and x_2, as described in Eqs. (1)-(4) (see Sections 5.1-5.2 in the Appendix for an explanation and derivations), and the correlation coefficient between *y*, Sahel rainfall as simulated in CMIP5, and its predicted values from regression model (6): $\hat{y} = ap_1 + bp_2$. Models highlighted in bold font are those whose value in the rightmost column is greater than 0.45, representing 20% of the total variance. Multi-model mean results are included in the last line of the table.

Table S2: same as in Table S1, but for the CMIP5 twentieth century/historical simulations. The second column from the left reports the ensemble size.

Table S3: same as in Table S1, but for the CMIP5 twenty-first century/RCP8.5 simulations. The second column from the left reports the ensemble size.

Table S1

CMIP5/PI-control pre-industrial	ρ	k	ϕ, arc °	$\lambda_2/(\lambda_1+\lambda_2)$, %	a	b	Correlation coefficient of y, $\hat{y}$
ACCESS1-0	0.188	0.946	53.18	40.23	-0.235	0.164	0.286
ACCESS1-3	0.228	1.027	41.66	38.55	-0.312	0.283	0.421
bcc-csm1-1	0.173	0.589	81.31	24.62	-0.004	-0.168	0.168
**BNU-ESM**	**0.317**	**1.078**	**38.33**	**33.75**	**-0.404**	**0.244**	**0.472**
CanESM2	0.357	0.939	49.99	31.92	-0.289	0.264	0.391
**CCSM4**	**0.479**	**1.162**	**36.25**	**25.17**	**-0.360**	**0.380**	**0.523**
**CESM1-CAM5**	**0.380**	**0.873**	**54.88**	**30.01**	**-0.207**	**0.610**	**0.644**
CMCC-CM	0.316	0.778	64.35	30.37	0.141	-0.047	0.149
CNRM-CM5	0.320	0.599	74.57	22.48	0.136	-0.083	0.159
CSIRO-Mk3-6-0	0.440	0.922	50.23	27.69	0.138	0.073	0.156
**FGOALS-g2**	**0.177**	**0.952**	**52.81**	**40.81**	**-0.244**	**0.448**	**0.511**
FIO-ESM	0.266	1.292	22.90	32.01	-0.051	-0.166	0.173
GFDL-CM3	0.460	0.951	48.10	26.87	0.358	0.067	0.364
GFDL-ESM2G	0.132	0.824	72.91	38.47	0.185	0.215	0.284
GFDL-ESM2M	0.348	1.156	33.66	31.33	-0.202	0.141	0.246
GISS-E2-H	-0.067	0.989	130.18	46.62	-0.155	0.042	0.160
GISS-E2-R	0.141	0.745	77.38	34.16	0.180	-0.188	0.261
**HadGEM2-CC**	**0.007**	**0.580**	**89.63**	**25.20**	**0.175**	**0.533**	**0.561**
**HadGEM2-ES**	**0.107**	**0.688**	**82.14**	**31.47**	**0.062**	**0.458**	**0.462**
inmcm4	0.300	0.888	55.85	33.95	-0.163	0.301	0.342
IPSL-CM5A-LR	0.423	0.666	67.32	22.59	0.078	0.250	0.262
IPSL-CM5A-MR	0.452	0.785	59.21	25.02	-0.097	0.426	0.437
IPSL-CM5B-LR	0.398	0.467	77.29	14.47	0.134	-0.264	0.297
MIROC-ESM	0.180	0.668	78.23	29.15	-0.040	0.229	0.232
**MIROC5**	**0.393**	**1.302**	**27.90**	**27.04**	**-0.533**	**0.229**	**0.580**
MPI-ESM-LR	0.357	1.074	39.36	31.82	-0.369	0.253	0.447
MPI-ESM-MR	0.342	0.857	57.17	31.45	-0.241	0.312	0.394
MRI-CGCM3	0.115	0.519	85.34	20.85	0.152	0.043	0.158
NorESM1-M	0.352	0.964	47.99	32.30	-0.110	0.197	0.226
Multi-model mean	*0.369*	*0.771*	*62.75*	*28.10*	*0.054*	*0.312*	*0.317*

Table S2

CMIP5/historical [1900-1999]	ens size	ρ	k	ϕ, arc °	$\lambda_2/(\lambda_1+\lambda_2)$, %	a	b	Correlation coefficient of y, $\hat{y}$
ACCESS1-0	1	0.303	0.735	67.97	29.22	-0.040	0.190	0.194
ACCESS1-3	1	0.299	0.908	53.96	34.36	-0.087	0.223	0.240
bcc-csm1-1	3	0.759	1.054	43.00	11.99	-0.015	0.289	0.290
BNU-ESM	1	0.553	1.144	38.13	21.80	-0.213	0.257	0.334
**CanESM2**	**5**	**0.758**	**1.186**	**38.63**	**11.72**	**-0.188**	**0.442**	**0.480**
**CCSM4**	**6**	**0.920**	**1.211**	**39.10**	**3.85**	**-0.367**	**0.488**	**0.610**
**CESM1-CAM5**	**3**	**0.621**	**1.086**	**41.19**	**18.78**	**-0.214**	**0.473**	**0.520**
CMCC-CM	1	0.322	1.027	42.60	33.85	-0.084	-0.112	0.140
CNRM-CM5	10	0.817	0.779	53.57	8.54	0.221	0.242	0.328
CSIRO-Mk3-6-0	10	0.765	0.884	49.59	11.56	-0.051	0.098	0.111
FGOALS-g2	5	0.881	1.150	40.48	5.83	-0.190	0.284	0.341
FIO-ESM	3	0.749	1.276	35.91	11.72	0.040	0.267	0.270
GFDL-CM3	4	0.732	0.847	51.41	13.00	0.297	0.334	0.447
GFDL-ESM2G	1	0.488	0.952	47.90	25.50	-0.244	0.300	0.386
GFDL-ESM2M	1	0.389	1.203	32.24	28.82	-0.192	-0.006	0.192
**GISS-E2-H**	**5**	**0.856**	**1.278**	**36.92**	**6.77**	**-0.334**	**0.422**	**0.538**
**GISS-E2-R**	**5**	**0.826**	**1.197**	**38.83**	**8.39**	**-0.316**	**0.360**	**0.479**
HadGEM2-CC	1	0.304	0.640	73.29	24.94	0.142	0.323	0.353
HadGEM2-ES	4	0.439	0.554	72.46	17.61	0.297	0.285	0.411
inmcm4	1	0.621	1.020	44.08	18.96	-0.180	0.135	0.225
**IPSL-CM5A-LR**	**5**	**0.901**	**0.806**	**51.80**	**4.72**	**-0.289**	**0.395**	**0.489**
**IPSL-CM5A-MR**	**1**	**0.675**	**0.982**	**45.77**	**16.23**	**-0.249**	**0.431**	**0.498**
IPSL-CM5B-LR	1	0.592	0.616	65.20	15.28	0.223	-0.180	0.286
**MIROC-ESM**	**3**	**0.462**	**1.065**	**41.11**	**26.75**	**-0.295**	**0.467**	**0.552**
**MIROC5**	**5**	**0.718**	**1.027**	**43.95**	**14.11**	**-0.228**	**0.593**	**0.635**
MPI-ESM-LR	3	0.826	0.833	51.29	8.40	0.072	0.373	0.380
MPI-ESM-MR	3	0.810	0.890	49.12	9.38	0.284	0.137	0.316
**MRI-CGCM3**	**3**	**0.615**	**0.670**	**61.89**	**15.81**	**0.403**	**0.365**	**0.544**
NorESM1-M	3	0.747	0.936	47.53	12.58	-0.162	0.248	0.296
Multi-model mean	***29***	***0.955***	***1.054***	***43.42***	***2.26***	***-0.320***	***0.477***	***0.574***

Table S3

CMIP5/RCP8.5 [2006-2099]	ens size	ρ	k	ϕ, arc °	$\lambda_2/(\lambda_1+\lambda_2)$, %	a	b	Correlation coefficient of y, $\hat{y}$
ACCESS1-0	1	0.974	1.104	42.09	1.27	0.186	0.196	0.270
ACCESS1-3	1	0.982	1.112	41.91	0.87	0.088	0.070	0.112
bcc-csm1-1	1	0.959	1.051	43.51	2.07	-0.101	0.142	0.174
**BNU-ESM**	**1**	**0.960**	**0.911**	**47.78**	**2.00**	**0.639**	**0.309**	**0.709**
CanESM2	5	0.994	1.069	43.07	0.29	0.173	0.236	0.292
**CCSM4**	**6**	**0.994**	**1.150**	**40.98**	**0.32**	**0.613**	**0.081**	**0.619**
CESM1-CAM5	3	0.991	1.173	40.42	0.41	0.188	0.392	0.435
CMCC-CM	1	0.978	1.031	44.12	1.08	-0.147	0.216	0.262
**CNRM-CM5**	**5**	**0.990**	**0.978**	**45.63**	**0.49**	**0.520**	**0.105**	**0.531**
**CSIRO-Mk3-6-0**	**10**	**0.997**	**1.089**	**42.54**	**0.13**	**-0.933**	**0.078**	**0.936**
**FGOALS-g2**	**1**	**0.976**	**0.683**	**55.89**	**1.04**	**0.707**	**0.169**	**0.727**
FIO-ESM	3	0.965	1.624	31.21	1.37	-0.134	0.121	0.181
**GFDL-CM3**	**1**	**0.984**	**0.935**	**46.97**	**0.82**	**0.607**	**0.259**	**0.660**
GFDL-ESM2G	1	0.951	0.948	46.62	2.45	-0.224	0.007	0.225
GFDL-ESM2M	1	0.945	0.975	45.76	2.76	-0.232	-0.025	0.233
GISS-E2-H	1	0.971	1.125	41.53	1.42	-0.314	0.305	0.438
**GISS-E2-R**	**2**	**0.973**	**1.236**	**38.82**	**1.30**	**-0.556**	**0.318**	**0.640**
HadGEM2-CC	3	0.978	1.074	42.90	1.11	0.252	0.300	0.391
HadGEM2-ES	4	0.989	1.118	41.76	0.56	-0.084	0.366	0.375
inmcm4	1	0.967	1.036	43.96	1.65	-0.001	0.085	0.085
IPSL-CM5A-LR	4	0.995	1.085	42.64	0.24	-0.089	0.293	0.306
IPSL-CM5A-MR	1	0.982	1.116	41.80	0.87	-0.051	0.225	0.231
IPSL-CM5B-LR	1	0.955	0.840	50.21	2.18	0.199	-0.127	0.236
**MIROC-ESM**	**1**	**0.973**	**0.939**	**46.86**	**1.36**	**0.636**	**0.123**	**0.648**
**MIROC5**	**3**	**0.963**	**0.989**	**45.33**	**1.87**	**0.450**	**0.622**	**0.768**
MPI-ESM-LR	3	0.988	1.028	44.20	0.59	-0.019	0.000	0.019
MPI-ESM-MR	1	0.965	1.064	43.16	1.76	0.201	0.347	0.401
MRI-CGCM3	1	0.962	0.927	47.25	1.90	0.241	0.158	0.288
**NorESM1-M**	**1**	**0.953**	**1.038**	**43.88**	**2.34**	**0.431**	**0.344**	**0.552**
Multi-model mean	***29***	***0.999***	***1.043***	***43.79***	***0.04***	***0.783***	***0.171***	***0.801***
